# Total Lisfranc dislocation without associated fracture

**DOI:** 10.11604/pamj.2023.45.164.22796

**Published:** 2023-08-17

**Authors:** Mohammed Hajjioui, Najia Hajjioui

**Affiliations:** 1 Traumatology and Orthopaedic Surgery, Military Hospital Moulay Ismail, Meknes, Morocco,; 2 Reeducation and Rehabilitation Medicine, Centre Hospitalier Universitaire de Fes, Fes, Morocco

**Keywords:** Lisfranc, dislocation, emergency

## Image in medicine

Lisfranc dislocation is a serious injury that it is important to recognize early and start treatment promptly because failure to recognize and treat these will lead to midfoot arthritis, chronic pain, and functional instability. An 18-year-old man was brought to the emergency department after a foot trauma, causing pain, swelling, and deformity of the foot. The dorsum of the foot was swollen and deformed with excruciating pain. The mechanism was direct due to high-energy direct, blunt force applied by a heavy object to the dorsum of the foot. The posterior tibial pulse was felt normally. Clinical diagnosis of tarsometatarsal dislocation was evocated. Radiologic findings revealed tarsometatarsal dislocation without fractures. Under anesthesia spine, a closed reduction in the emergency was performed in the axis of the first metatarsal to dislodge its mortise base between the medial and lateral cuneiform with control under an image intensifier by pulling. Post-reduction X-rays confirmed reduction. The foot and ankle were immobilized for six weeks with a complete cast followed by rehabilitation and the functional result was good after a year of decline. In this case, we emphasize the importance of not missing out on the diagnosis and the importance of treating this lesion urgently to avoid complications.

**Figure 1:**
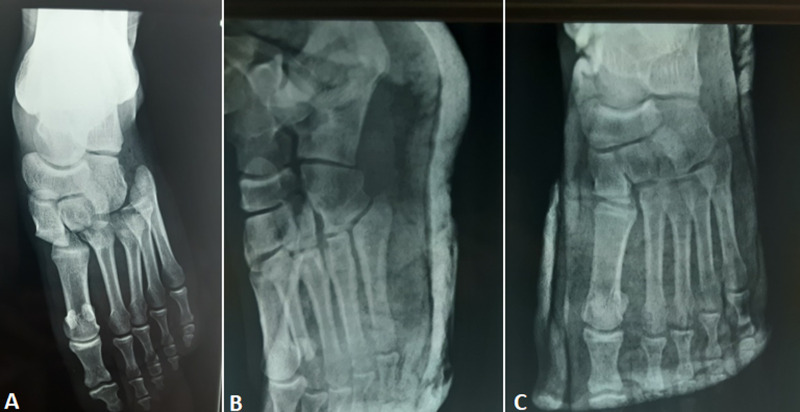
A) foot X-ray with Lisfranc dislocation; B, C) radiological control after reduction

